# Hierarchically Assembled Type I Collagen Fibres as Biomimetic Building Blocks of Biomedical Membranes

**DOI:** 10.3390/membranes11080620

**Published:** 2021-08-12

**Authors:** Jie Yin, David J. Wood, Stephen J. Russell, Giuseppe Tronci

**Affiliations:** 1 Clothworkers’ Centre for Textile Materials Innovation for Healthcare, School of Design, University of Leeds, Leeds LS2 9JT, UK; jie.yin@henkel.com (J.Y.); s.j.russell@leeds.ac.uk (S.J.R.); 2 Biomaterials and Tissue Engineering Research Group, School of Dentistry, St. James’s University Hospital, University of Leeds, Leeds LS9 7TF, UK; d.j.wood@leeds.ac.uk

**Keywords:** type I collagen, photoactive, wet spinning, sequential functionalization

## Abstract

Wet spinning is an established fibre manufacturing route to realise collagen fibres with preserved triple helix architecture and cell acceptability for applications in biomedical membranes. However, resulting fibres still need to be chemically modified post-spinning to ensure material integrity in physiological media, with inherent risks of alteration of fibre morphology and with limited opportunities to induce fibrillogenesis following collagen fixation in the crosslinked state. To overcome this challenge, we hypothesised that a photoactive type I collagen precursor bearing either single or multiple monomers could be employed to accomplish hierarchically assembled fibres with improved processability, macroscopic properties and nanoscale organisation via sequential wet spinning and UV-curing. In-house-extracted type I rat tail collagen functionalised with both 4-vinylbenzyl chloride (4VBC) and methacrylate residues generated a full hydrogel network following solubilisation in a photoactive aqueous solution and UV exposure, whereby ~85 wt.% of material was retained following 75-day hydrolytic incubation. Wide-angle X-ray diffraction confirmed the presence of typical collagen patterns, whilst an averaged compression modulus and swelling ratio of more than 290 kPa and 1500 wt.% was recorded in the UV-cured hydrogel networks. Photoactive type I collagen precursors were subsequently wet spun into fibres, displaying the typical dichroic features of collagen and regular fibre morphology. Varying tensile modulus (*E* = 5 ± 1 − 11 ± 4 MPa) and swelling ratio (*SR* = 1880 ± 200 − 3350 ± 500 wt.%) were measured following post-spinning UV curing and equilibration with phosphate-buffered saline (PBS). Most importantly, 72-h incubation of the wet spun fibres in PBS successfully induced renaturation of collagen-like fibrils, which were fixed following UV-induced network formation. The whole process proved to be well tolerated by cells, as indicated by a spread-like cell morphology following a 48-h culture of L929 mouse fibroblasts on the extracts of UV-cured fibres.

## 1. Introduction

Fibrous materials play a crucial role in the design of biomedical membranes, with applicability in healthcare spanning wound care [1] and infection control [2] up to tendon repair [3] and treatment for intervertebral disc degeneration [4]. For healthcare products based on synthetic polymers, process controllability at microscopic and macroscopic scales enables variation in fibre diameter, fabric pore size and three-dimensional assembly [5]. However, the introduction of biomimetic cues at the molecular/nanoscale may be required for some healthcare applications to ensure cell migration and proliferation, tissue integration in vivo and immune regulation [6]. In contrast to synthetic polymers, customisation of biomimetic systems into fibrous materials presents challenges with regards to fibre spinning compatibility, given the limited solubility in organic solvents, the long-range nanoscale organisation and the shape instability of resulting fibres in aqueous environments [7]. While bespoke manufacturing routes have been developed to enable the conversion of biomimetic systems into fibres [8,9], the molecular manipulation of biopolymers derived from the extracellular matrix (ECM) of biological tissues may offer a more time effective approach to ensure the formation of fibres with biomimetic features from the molecular up to the macroscopic scale, as well scalability for industrial uptake [10,11].

Collagen is the main protein component of the ECM and plays key roles in the properties and functions of biological tissues [12]. This is largely attributed to its unique hierarchical organisation in vivo, involving the assembly of three left-handed polyproline-II helices into a right-handed triple helix [13,14,15]. The high content of glycine, proline and hydroxyproline in the polyproline-II helices is largely responsible for the stabilisation of the collagen triple helix via inter-chain hydrogen bonds. Collagen triple helices are folded into aligned fibrils in vivo, whereby the collagen triple helices are staggered by the characteristic 67 nm D-band spacing. These aggregates are fixed by covalent crosslinks, whose density increases with ageing, and which involve covalent bonds between either two lysines or a lysine and arginine sidechains. Recapitulating such complex structures in vitro is an ongoing challenge given the different limitations dictated by each aggregation state with regards to e.g., scalability, chemical accessibility, and controlled structure-property relations [16,17]. To align research development with industrial demands, extraction of collagen from biological tissues has been pursued to accomplish either triple helices (monomeric collagen) or single polyproline-II helices (i.e., gelatin), which are non-crosslinked and therefore soluble in aqueous environments.

After collagen extraction, a range of manufacturing and synthetic crosslinking routes have been applied to generate water-insoluble collagen fibres with preserved material integrity in biological conditions and biomimetic features at the molecular and microscale, e.g., cell-binding sequence and collagen fibril-like fibre diameters [18,19,20]. Wet spinning has proven to be a particularly successful method to produce individual collagen fibres [21] and multifilament yarns [22] of collagen triple helices, enabling further generation of fibrous assemblies and three-dimensional macroscopic fabrics. At the same time, crosslinking of collagen post-spinning is still associated with issues with respect to the safety and macroscopic properties of the resulting products [23]. Indeed, current synthetic crosslinking methods are typically carried out in the wet state, generating risks of uncontrollable fibre swelling and alteration of fibre morphology during the crosslinking. Furthermore, post-spinning fibre fixation in the crosslinked state prevents the possibility of inducing the folding of collagen triple helices into fibrils (fibrillogenesis) in physiological conditions, thereby limiting the long-range recapitulation of collagen structures. Ultimately, current crosslinking procedures typically involve either potentially toxic compounds, e.g., glutaraldehyde [24], or relatively harsh dehydrothermal treatments (110 °C, 5 days) [25,26], raising issues with regards to the fibre acceptability in the biological environment as well as the retention of collagen hierarchical organisation.

We have reported the design of photoactive type I collagen systems and respective fabrication of photoinduced collagen networks [27], as a means to circumvent the above-mentioned challenges associated with currently available crosslinking methods, so that mechanical properties could be adjusted at both micro- and macroscale [28]. Covalent coupling of single photoactive residues also offers an opportunity to generate improved material processability in water, due to the secondary interactions mediated by introduced residues at the molecular scale [29]. These synthetic strategies may also be associated with inherent drug-free pharmacological effects in physiological conditions, such as the chelation and inhibition of proteolytic enzymes [30,31], which could be exploited to restore tissue homeostasis [32]. Aforementioned features are therefore key when aiming to accomplish rapid customisation of macroscopic properties “on-demand” with minimal risks of fibre alteration, and may also offer an additional experimental dimension to stabilise long-range refolded collagen structures induced via fibre conditioning in physiological conditions prior to photocuring.

This work aims to investigate whether photoactive type I collagen precursors could be wet spun into mechanically stable fibres and fixed in a controlled network architecture following UV curing, to accomplish biomimetic features from the molecular up to the macroscopic scale. We hypothesised that covalent functionalisation of collagen lysines with either single or multiple photoactive monomers minimises risks of fibre dissolution in water both prior to or following network formation, so that hierarchically assembled fibres made of either covalently crosslinked triple helices or renatured collagen fibrils could be accomplished. Our strategy was therefore to first investigate the structure-property relations of UV-cured hydrogels made of three distinct photoactive collagen precursors, and subsequently to customise these in wet spun fibres investigating the effects of fibre conditioning and UV curing on macroscopic properties and collagen short/long-range organisation.

## 2. Materials and Methods

### 2.1. Materials

4-vinylbenzyl chloride (4VBC), glycidyl methacrylate (GMA), methacrylic anhydride (MA), triethylamine (TEA), 2-Hydroxy-4′-(2-hydroxyethoxy)-2-methylpropiophenone (I2959), 2,4,6-trinitrobenzenesulfonic acid (TNBS), acetic acid (AcOH), and Dulbecco’s Phosphate Buffered Saline (PBS) were purchased from Sigma Aldrich (St. Louis, MO, USA) and used as received. Rat tails were received from Central Biomedical Services at the University of Leeds (UK) and used for the acidic extraction of type I collagen. Dulbecco’s modified Eagle’s medium (DMEM), foetal calf serum (FBS) and penicillin-streptomycin (PS) were purchased from Gibco (Waltham, MA, USA). All the other chemicals were purchased from Sigma Aldrich (St. Louis, MO, USA).

### 2.2. Extraction of Type I Collagen

Type I collagen was extracted in-house from rat tails [27]. Briefly, frozen rat tails were thawed in distilled water for about 15 min. Individual tendons collected from each rat tail were pulled out of the tendon sheath and incubated in 50 mL of 17.4 mM AcOH solution at 4 °C. After three days, the supernatant was centrifuged at 10,000 r·min^−1^ for 30 min, and the mixture was freeze-dried to obtain type I collagen.

### 2.3. Sequential Functionalisation of Rat Tail Collagen

In-house extracted collagen from rat tails (CRT) was solubilised (0.25 wt.%) in 10 mM hydrochloric acid (HCl) via magnetic stirring at room temperature. Solution pH was neutralized to pH 7.4 prior to the addition of 1 wt.% of polysorbate-20 (with respect to the initial weight of the collagen solution). 4VBC was introduced in the reacting mixture with a 30-molar ratio with respect to the molar content of collagen lysines (~3 × 10^−4^ mol·g^−1^, determined via TNBS analysis), followed by the addition of an equimolar content of TEA ([TEA] = [4VBC]). After a 24-h reaction time, MA and TEA were added ([MA] = [4VBC]; [TEA] = [MA]) and the reaction was carried out for another 24 h, prior to precipitation in a 10-fold excess of pure ethanol. The 4VBC- and MA-reacted CRT product was collected by centrifugation and the pellet air-dried. Functionalised controls were also prepared via a single reaction of CRT with either GMA or 4VBC, using a 30-molar excess of each monomer and a 30-molar excess of TEA. These reacting mixtures were precipitated in ethanol, centrifuged and the pellets air-dried as previously outlined.

### 2.4. Chemical Characterisation of Photoactive Collagen Precursors

2,4,6-trinitrobenzenesulfonic acid (TNBS) colourimetric assay was employed to determine the molar content of free amino groups and the degree of functionalisation of collagen [27,28].

Circular dichroism (CD) spectra of native, functionalised and wet spun collagen were acquired with a Jasco J-715 spectropolarimeter by dissolving the collagen material (0.2 mg∙mL^−^^1^) in 10 mM HCl [12]. Sample solutions were collected in quartz cells of 1.0 mm path length, and CD spectra were obtained with 2 nm bandwidth and 20 nm∙min^−^^1^ scanning speed. The spectrum of the 10 mM HCl control solution was subtracted from each sample spectrum.

The viscosity of the functionalised collagen solutions (6 mg·mL^−1^) was measured using a benchtop Brookfield DV-E Viscometer (Brookfield Engineering Laboratories, Inc., Middleboro, MA, USA). Rotating spindle s34 was employed and measurements were recorded at room temperature at a rotating speed of 30 rpm [21].

### 2.5. Fabrication of UV-Cured Hydrogels and Wet Spun Fibres

For the fabrication of UV-cured hydrogels, sequentially-reacted and 4VBC-reacted CRT precursors were magnetically stirred at 4 °C in a 17.4 mM AcOH solution containing 1 wt.% of I2959 (as a water-soluble photoinitiator) to reach a final collagen concentration (*c*) of either 4 or 8 mg∙mL^−1^. Rather than the 17.4 mM AcOH solution, GMA-functionalised CRT was dissolved in an I2959-supplemented PBS solution (1 wt.% I2959, 10 mM PBS, pH 7.4) as outlined above. The resulting photoactive solutions were cast onto a 24-well tissue culture plate, followed by UV irradiation (Spectroline, 365 nm, Melville, NY, USA) for 30 min on top and bottom sides. Formed hydrogels were thoroughly washed in distilled water to remove unreacted compounds. Samples were air-dried following dehydration via an ascending series of distilled water-ethanol mixtures (0–100% ethanol).

For the fabrication of wet spun fibres, the reacted CRT products were dissolved (*c* = 6 mg·mL^−1^) in 17.4 mM AcOH via magnetic stirring at room temperature. The CRT solutions were transferred into a 10 mL syringe equipped with a needle of 1.1 mm internal diameter. The solution-containing syringes were loaded on a syringe pump, so that wet spinning was carried out at room temperature with a dispensing rate of 12.5 mL·h^−1^ and the syringe tip submerged in a coagulation bath containing pure ethanol. Individual fibres were air-dried and subsequently incubated (20 min) in an aqueous solution of ethanol (90 wt.% EtOH) supplemented with I2959 (1 wt.%). UV irradiation (Spectroline, 365 nm, Melville, NY, USA) was carried out for 30 min and the UV-cured fibres were washed with pure ethanol and then air-dried.

### 2.6. Hydrolytic Degradation Tests

UV-cured networks (*n* = 3) of known dry mass (*m_d_*) were individually incubated in vials containing 20 mL of PBS (10 mM, pH 7.4, 37 °C) for 75 days, with medium changed every three days. At selected time points, samples were collected, rinsed with distilled water, air dried, and weighed (*m_t_*). The relative mass (*µ_rel_*) was calculated via Equation (1):

(1) $$\mu_{rel}=\frac{m_{t}}{m_{d}}\times 100$$

A linear regression analysis of the averaged data points was performed (y = a + bx) and the linear curve was included in the data plot.

### 2.7. Fibre Conditioning and Transmission Electron Microscopy (TEM)

Dry wet spun fibres were incubated in PBS at 37 °C for up to 72 h to induce collagen fibrillogenesis. Samples were subsequently immersed (20 min) in an aqueous solution of ethanol (90 vol.% EtOH) supplemented with I2959 (1 wt.%), followed by 30-min UV irradiation (Spectroline, 365 nm). The resulting UV-cured fibres were washed in ethanol before air drying. Bundles of 10 individual UV-cured fibres were tied at both ends with 5-0 polyamide suture and hydrated for 1 h in PBS, adapting a previously reported protocol [24]. The samples were stained for 1 h with 1 wt.% osmium tetroxide in PBS, washed (30 min, ×2) with PBS and dehydrated in distilled water-ethanol mixtures (20, 40, 60, 80, 2 × 100 vol.%). Fibres were then resin embedded and polymerised (24 h, 60 °C). Using an ultramicrotome (Reichert-Jung, Ultracut E, Munich, Germany) and a diamond knife, ultrathin sections (80–100 nm) were cut to reveal the axial perspective of the fibre core. Following post-staining with saturated uranyl acetate and Reynold’s stain, sections were examined by TEM in a JEOL JEM 1400 (Tokyo, Japan) with a tungsten filament running at 120 kV.

### 2.8. Gel Content and Swelling Ratio Tests

Freshly-synthesised, non-washed hydrogels (*n* = 3) were frozen at 80 °C and then lyophilized. The dry weight (*m_d_*) of the resulting crosslinked network was recorded. The dry samples were then incubated in 17.4 mM AcOH for 48 h at RT, prior to sample collection, lyophilisation and weight measurement (*m*_0_). The gel content (*G*) was determined according to Equation (2):

(2) $$G=\frac{m_{0}}{m_{d}}$$

Together with the gel content, swelling tests were performed on the dry UV-cured networks and dry UV-cured wet spun fibres (1 mg of randomly twisted fibres) via 24-h incubation in PBS at 37 °C (*n* = 3). The PBS-equilibrated samples were paper blotted and weighed to obtain the swollen sample weight (*m_s_*). The swelling ratio (*SR*) was calculated via Equation (3):

(3) $$SR=\frac{m_{s}-m_{d}}{m_{d}}\times 100$$

where *m_s_* and *m_d_* represent the weights of the swollen and dry samples, respectively. Data were presented as mean ± SD.

### 2.9. Mechanical Property Characterisation

Water-equilibrated hydrogel discs (Ø = 12 mm) were compressed at room temperature with a compression rate of 3 mm·min^−1^ and a 200 N load cell (Zwick Roell Z010). Compression was carried out up to sample break. The stress at break (*σ_b_*) and compression at break (*ε_b_*) were recorded, while the compressive modulus (*E_c_*) was calculated by fitting (*R*^2^ ≥ 0.98) the linear region (*ε*: 30–40%) of the stress-strain curve. Six replicates were used for the UV-cured hydrogels made of CRT functionalised with both 4VBC and MA residues.

The dry wet spun collagen fibres were tested in tension (Zwick Roell Z010) using a 10 N load cell, a rate of extension of 0.03 mm·s^−1^ and a gauge length of 5 mm. The testing of dry fibres was carried out in a controlled environment with a room temperature of 18 °C and relative humidity of 38%. A total of 8–10 individual fibres were tested from each group and SEM was used to measure the fibre diameter. To measure the tensile modulus and strength in hydrated conditions, the wet spun fibres were immersed in PBS at 37 °C for 24 h; optical microscopy was subsequently used to measure the diameter of these hydrated fibres. Tensile testing was operated with an extension rate of 1 mm·s^−1^ to minimise risks of fibre dehydration. Measurements were reported as mean ± SD and the elastic modulus was obtained by a linear regression fit.

### 2.10. Chemical, Thermal and Structural Analysis

Attenuated total reflectance Fourier transform infrared (ATR-FTIR) spectroscopy was performed on dry wet spun fibres using a Perkin-Elmer Spectrum BX (Waltham, MA, USA) over a range of 800–4000 cm^−1^ at a resolution of 2 cm^−1^.

Differential Scanning Calorimetry (DSC) was employed to investigate the thermal denaturation of collagen samples (TA Instruments Thermal Analysis 2000 System and 910 Differential Scanning Calorimeter cell base, New Castle, DE, USA). DSC temperature scans were conducted in a temperature range of 10–100 °C using a 10 °C·min^−1^ heating rate. Next, 5–10 mg of wet sample (*n* = 2) was applied in each measurement and two scans were used for each sample formulation. The DSC cell was calibrated using indium and zinc with a 10 °C·min^−1^ heating rate under 30 cm^3^·min^−1^ of nitrogen atmosphere.

Wide-angle X-ray scattering (WAXS) was carried out on dry collagen networks with a Bruker D8 Discover (40 kV, 30 mA, l = 0.154 nm). The detector was set at a distance of 150 mm covering 2*θ* from 3° to 40°. The collimator was 2.0 mm wide, and the exposure time was 10 s per frame. Collected curves were subtracted from the background (no sample loaded) curve and fitted with polynomial functions (*R*^2^ > 0.93).

### 2.11. Scanning Electron Microscopy

The surface morphology of wet spun fibres was inspected via scanning electron microscopy (SEM, Hitachi SU8230, Tokyo, Japan). Samples were gold-sputtered (Bioz, JFC-1200, Los Altos, CA, USA) and visualised with a beam intensity of 10 kV.

### 2.12. Extract Cytotoxicity Tests

An extract cytotoxicity assay was conducted with L929 cells via qualitative cell morphology observations. First, 1 mg of UV-cured wet spun fibres was disinfected with 70 vol.% EtOH for 30 min followed by 15-min UV light exposure. Samples were subsequently incubated in PBS for 30 min, sealed in a sterile centrifuge tube and weighed. Sample extracts were prepared by incubating (37 °C, 72 h) the wet samples in completed DMEM (10 vol.% FCS, 1 wt.% PS) (10 μL completed DMEM per μg of the hydrated sample). L929 mouse fibroblasts (10^5^ cells·mL^−1^) were seeded onto a 96-well plate (100 μL cell suspension per well) for 24 h to enable cell confluence. Subsequently, the cell culture medium was replaced with sample extract into each well and cells cultured for another 48 h. Eight replicas were used for each sample group. Transmitted light microscopy was used to observe the cell morphology.

## 3. Results and Discussion

**Figure 1:**
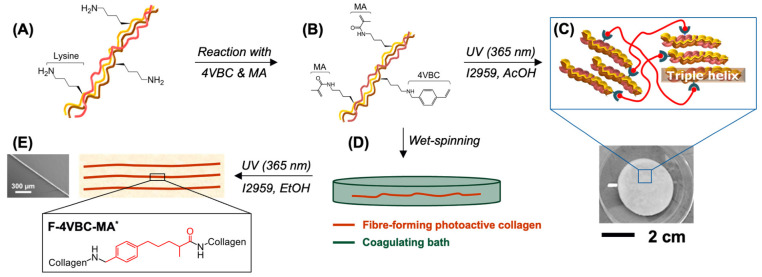
Design of hierarchically assembled type I collagen fibres. Reaction of CRT (**A**) with 4VBC and MA enables covalent coupling of multiple photoactive residues (**B**). UV-curing generates a covalent hydrogel network following solubilisation of the photoactive CRT precursor in the presence of the I2959 photoinitiator (**C**). The photoactive CRT precursor is wet spun against ethanol (**D**) and UV-cured to realise individual fibres with enhanced material integrity and controlled nanoscale organisation (**E**).

Collagen was extracted in-house from rat tails as an inexpensive source of type I collagen raw material. Covalent functionalisation of CRT was pursued with either single or multiple ethylenically unsaturated monomers, i.e., 4VBC, MA and GMA, to generate varied photoactive precursors and UV-cured networks with customised micro-/macroscopic format and protein configuration (Figure 1). The effect of covalent functionalisation, wet spinning route and UV curing were investigated both in terms of macroscopic properties and collagen organisation, aiming to build hierarchically assembled fibres with biomimetic features across length scales. 

Samples of photoactive collagen were coded as either 4VBC-MA, 4VBC or GMA, whereby 4VBC-MA indicates a collagen precursor functionalised with both 4VBC and MA; whilst 4VBC and GMA refer to collagen precursors functionalised with either 4VBC or GMA, respectively. Samples of UV-cured hydrogel networks are identified as either 4VBC-MA*, 4VBC* or GMA*, whereby * indicates the crosslinked state. Samples of wet spun fibres and UV cured wet spun fibres are labelled as F-4VBC-MA, F-4VBC, F-GMA and F-4VBC-MA*, F-4VBC*, F-GMA*, where F and * identify the fibrous and crosslinked state, respectively. Ultimately, the wet spun fibre control made of native CRT is coded as F-C.

### 3.1. Synthesis of Photoactive Collagen Precursors

Two CRT precursors were obtained via reaction with either 4VBC (an aromatic, stiff monomer) or GMA (an aliphatic, flexible monomer). Both 4VBC and GMA residues proved to be coupled to the lysine terminations of respective collagen products, so that the averaged degrees of functionalisation (*F*) of 45 mol.% and 65 mol.% were respectively observed via TNBS assay (Table 1). In an effort to customise the collagen macroscopic properties via variation of covalently coupled monomers, an additional collagen product was realised via a one-pot sequential reaction with both 4VBC and MA, whereby MA was selected as a methacrylate monomer of known high reactivity towards collagen [28,29].

Accordingly, nearly complete consumption of free amino groups of CRT was measured in resulting samples, corresponding to an averaged *F* of over 90 mol.% and an averaged MA content of 52 mol.% (Table 1). All functionalised CRT samples revealed typical dichroic patterns of type I collagen, whereby the intensity of the triple helix-related positive peak was observed to be inversely related to the degree of collagen functionalisation (Supplementary Figure S1), in line with the consumption of triple helix-stabilising lysine terminations.

### 3.2. Macroscopic Characterisation of UV-Cured Hydrogels

**Figure 2:**
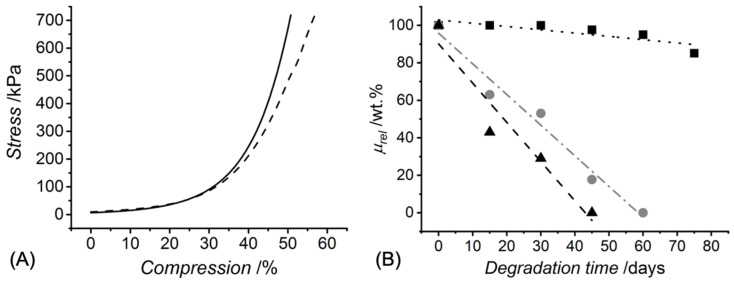
(**A**): stress-compression curves of hydrogels 4VBC-MA* prepared with either 0.4 (**– –**) or 0.8 wt.-% (**—**) concentration of sequentially-functionalised CRT. (**B**): relative mass profiles displayed by UV-cured hydrogels 4VBC-MA* (■, **∙∙∙**: y = 102.85 − 0.18x, *R*^2^ = 0.71), 4VBC* (●, **—·—**: y = 95.8 − 1.64x, *R*^2^ = 0.98), and GMA* (▲, **– –**: y = 90.1 − 2.09x, *R*^2^ = 0.93) during hydrolytic degradation in vitro (37 °C, pH 7.4).

UV exposure of the functionalised CRT products in the presence of the I2959 photoinitiator promptly led to complete gelation, whereby the effect of *c* and type of functionalisation were explored (Table 1). A value of *G* of at least 90 wt.% was measured in UV-cured networks prepared with increased CRT concentration (*c* = 0.8 wt.%). Hydrogels deriving from single collagen functionalisation appeared to display an increased value of *G* compared to sequentially functionalised variants, an observation that may be linked to the increased viscosity of UV-curing mixtures containing 4VBC-functionalised (*η* = 1156 mPa·s, *c* = 0.6 wt.%) with respect to GMA-functionalised (*η* = 170 mPa·s, *c* = 0.6 wt.%) samples. On the other hand, much lower *G* values were recorded when hydrogels 4VBC-MA* were prepared with a decreased collagen concentration (*c*: 0.8 → 0.4 wt.%), indicating that the molar content of photoactive groups in the UV-curing system directly affects the gelation yield. The concentration-induced variation of crosslink density was further corroborated by the variation of macroscopic properties, whereby an averaged increase in SR (900 → 1560 wt.%) was measured in hydrogel 4VBC-MA* prepared with decreased *c*, in line with the corresponding decrease in *G*, whilst insignificant variations in *E_c_*, *ε_b_* and *σ_b_* were observed (Table 1 and Figure 2A).

Other than the variation in *c*, broader adjustments in *SR* and compression properties were observed in hydrogels prepared from CRT precursors with different types and extents of functionalisation (*c* = 0.8 wt.%, Table 1). Sequentially functionalised CRT networks revealed the lowest averaged *SR* (900 wt.%) followed by samples 4VBC* (*SR* = 1965 wt.%) and GMA* (*SR* = 1262 wt.%), reflecting the nearly complete crosslinking of the collagen lysines. The introduction of 4VBC residues was found to generate stronger hydrogels even in the case of single (and decreased) collagen functionalisation, correlating with the molecular rigidity of the corresponding aromatic crosslinking segment, in contrast to the more flexible methacrylate crosslinking segments [27,28].

The extent of collagen functionalisation was proven to directly affect the hydrolytic degradability of the corresponding hydrogels, so that samples 4VBC-MA* retained about 85 wt.% of the original mass during a 75-day incubation in PBS, in contrast to the complete dissolution observed with samples 4VBC* (after 60 days) and GMA* (after 40 days) (Figure 2B). Linear fitting of the single data points supported a surface erosion degradation mechanism for all hydrogel samples [33], in agreement with the high *SR* measured in the freshly synthesised samples (Table 1) and the absence of an induction period typically observed in bulk-degrading polymers [34]. Interestingly, the faster degradation of UV-cured GMA- compared to 4VBC-functionalised samples seemed inversely related to the variations in the *F* observed in respected CRT precursors. The most logical explanation for the above degradation profiles is the different hydrolytic reactivity of the amide bonds formed following covalent coupling of either 4VBC or GMA, and the development of additional 4VBC-mediated secondary interactions during the degradation of network 4VBC*.

These results, therefore, indicate that both the type and the degree of functionalisation are key to accomplishing customised macroscopic properties of UV-cured CRT networks, so that a controlled structure-property relationship can be developed.

### 3.3. Thermal Properties and Structural Analysis of UV-Cured Hydrogels

**Figure 3:**
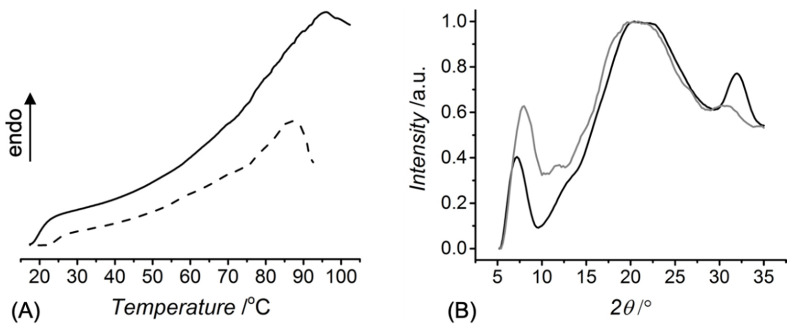
(**A**): DSC thermograms of hydrogels 4VBC-MA* prepared with either 0.4 (**–·–**) or 0.8 wt.-% (**—**) concentration of the sequentially-functionalised CRT precursor. (**B**): Typical WAXS spectra of the dry collagen network 4VBC-MA* (**—**) and native CRT control (**—**).

DSC and WAXS analyses were subsequently carried out to investigate the thermal properties and structural organisation of CRT in the crosslinked state (Figure 3). The thermal properties of type I collagen are described by the denaturation temperature (*T_d_*) of triple helices, above which unfolding into randomly coiled polypeptides occurs. The *T_d_* of CRT has been recorded in the range of 60–70 °C, whereby covalent crosslinking highly affects the thermal stability of collagen [20,21,27]. DSC thermograms of the UV-cured CRT hydrogels indicated an endothermic transition at 86–96 °C, which is attributed to the denaturation of collagen triple helices into random coils [20,22,35]. Variations of sample 4VBC-MA concentration (*c* = 0.4 → 0.8 wt.%) in respective hydrogel-forming solutions were found to directly impact on the *T_d_* (86 → 96 °C) of each resulting hydrogel (Figure 3A), in line with the concentration-induced effect on the crosslink density and thermal stabilisation of triple helices [20,22,23].

To further explore the CRT organisation in the crosslinked state, WAXS measurements were performed on the dry UV-cured networks. The WAXS spectrum of type I rat tail collagen displays three main peaks [17,20,28,36], identifying the intermolecular lateral packing of collagen triple helices (*d* ~ 1.1 nm, 2*θ* ~ 8°), the isotropic amorphous region (*d* ~ 0.5 nm, 2*θ* ~ 20°) and the axial periodicity (*d* ~ 0.29 nm, 2*θ* ~ 31°) of polypeptide subunits along a single polyproline-II chain (Figure 3B). The aforementioned peaks were detected in the spectrum of CRT network 4VBC-MA*, whereby the triple helix-related diffraction peak (2*θ* ~ 8°) was found at a slightly lower angle compared to the case of the CRT sample, while the peak associated with the single polyproline-II helices was recorded at an increased angle. These observations confirm the presence of collagen triple helices in the crosslinked state, although with an increased lateral triple helix packing [36] and decreased axial periodicity with respect to the native CRT configuration. This result correlates with the introduction of bulky 4VBC residues on the CRT backbone.

Moreover, sample 4VBC-MA* revealed a significantly reduced integration ratio (*X_c_* = 0.08) between the triple helix-related and amorphous regions with respect to the native CRT sample (*X_c_* = 0.15). The aforementioned variation in integration ratio supports previous CD data on the functionalised CRT precursors (Supplementary Figure S1), whereby an inverse relationship was found between the *F* and respective triple helix content.

### 3.4. Fabrication of Wet Spun Fibres of Photoactive Collagen and UV-Cured Networks

Following characterisation of the UV-cured hydrogels at the molecular and macroscopic scale, attention turned to the customisation of the aforementioned UV-cured hydrogel networks into fibres, whereby wet spinning was selected as a triple helix-compliant route to generate individual collagen fibres. A solution of AcOH (17.4 mM, pH ~ 3) was selected for the solubilisation of the functionalised CRT precursors and preferred over other acidic solutions (e.g., 10 mM HCl, pH ~ 2). The slightly higher pH was leveraged to control the secondary interactions between amino acidic terminations and respective solution viscosity to ensure continuous wet spinning and minimise risks of nozzle blockage. Solutions of 4VBC-functionalised collagen prepared with an intermediate concentration compared to the one used for the formation of the UV-cured network (Table 1) presented a slightly higher viscosity (*η* = 1156 mPa·s, *c* = 6 mg·mL^−1^) compared to the one measured in native CRT solutions with increased concentration (*c* = 8 mg·mL^−1^, [21]) and higher than the one of solutions of GMA-functionalised CRT (*η* = 170 mPa·s, *c* = 0.6 wt.%). These observations are attributed to the presence of rigid 4VBC residues in the CRT backbone and they are capable of inducing additional secondary interactions [29], similarly to the effect of branching chains observed in solutions of polymer-grafted collagen [35].

**Figure 4:**
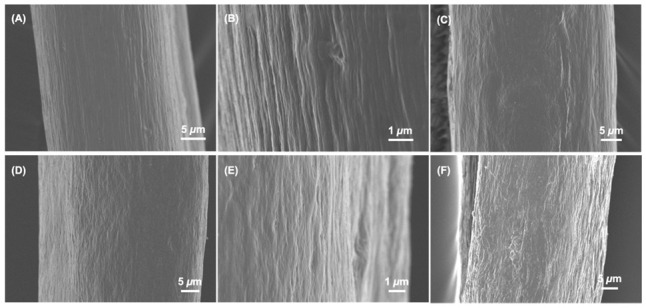
SEM of individual wet spun (**A**,**B**,**D**,**E**) and UV-cured (**C**,**F**) fibres prepared from either 4VBC-functionalised CRT (**A**–**C**) or GMA-functionalised CRT (**D**–**F**). (**A**,**B**): F-4VBC; (**C**): F-4VBC*. (**D**,**E**): F-GMA; (**F**): F-GMA*.

Despite the above significant variations in solution viscosity, micron-scale wet spun fibres were successfully obtained with a continuous surface morphology, as confirmed via SEM (Figure 1E and Figure 4A,D). Wet spinning was carried out in ethanol as a typical non-solvent for collagen, aiming to minimise the need for additional washing steps and the risk of swelling-induced fibre alteration, associated with aqueous coagulating baths [24]. Such an approach was also consistent with producing wet spun fibres of increased density [37]. Samples F-4VBC (Ø = 54 ± 5 μm), F-GMA (Ø: 39 ± 10) and F-4VBC-MA (Ø = 51 ± 5 μm) displayed significantly lower fibre diameters with respect to the sample of F-C (Ø = 80 ± 3 μm) (Table 2), a result that agrees with previous trends in solution viscosity and with the effect of solution viscosity on fibre diameter [7,10,35]. Higher resolution images revealed the presence of grooves on the surface of both sample F-4VBC (Figure 4B) and F-GMA (Figure 4E), which may be attributed to the shear stress generated during wet spinning in light of the relatively high solution viscosity [21,36]. Nevertheless, the aforementioned values of fibre diameter are comparable to previously reported wet spun collagen fibres [38,39,40], indicating that the covalent functionalisation introduced to the CRT backbone at the molecular scale does not significantly affect the fibre microscale.

Together with the characterisation of the fibre morphology, CD spectra of respective wet spun fibres confirmed the typical dichroic patterns of rat tail collagen (Supplementary Figure S1), consistent with the presence of native CRT rather than gelatin [27,28], and supporting previous DSC and WAXS analysis (Figure 3). While decreased intensities were observed for both triple helix-related (positive) and polyproline-II-related (negative) signals, the above-mentioned CD results highlight the collagen-compliant nature of the presented wet spinning route, enabling a degree of triple helix preservation in the resulting fibres together with the covalent coupling of multiple photoactive residues.

Additional confirmation of the chemical composition of the wet spun fibres was also obtained by ATR-FTIR spectroscopy (Supplementary Figure S2), indicating the main bands of native CRT, i.e., the amide I band at 1640 cm^−1^, resulting from the stretching vibrations of peptide C = O groups; the amide II band at 1540 cm^−1^, deriving from N–H bending and C–N stretching vibrations; the amide III band centred at 1240 cm^−1^, assigned to the C–N stretching and N–H bending vibrations from amide linkages, as well as wagging vibrations of CH_2_ groups in the glycine backbone and proline side chains [10,20,21,27]. Other than CRT-related signals, a shoulder at about 1636 cm^−1^ was detected, which provides evidence of the presence of 4VBC- and MA-related vinyl groups in the CRT backbone, despite the masking effect of previously mentioned amide bands of CRT.

Having confirmed their morphology and chemical composition, UV curing of wet spun fibres was subsequently carried out in an I2959-supplemented aqueous solution of ethanol (90 wt.% EtOH, 1 wt.% I2959). The presence of water in the UV-curing medium was pursued to enable uptake of the photoinitiator in the fibre, thereby promoting covalent crosslinking of the CRT molecules present in the fibre core rather than the ones located on the fibre surface. UV-irradiated samples demonstrated a retained wet spun fibre morphology (Figure 4C,F), whereby minimal variations in fibre diameter (Ø = 40 ± 10–51 ± 10 µm) were measured with respect to non-cured samples (Table 2). This observation therefore indirectly supports the beneficial effect of the covalently coupled photoactive residues aiming to accomplish enhanced wet-state material processability and wet-state integrity with respect to wet spun fibres made of native type I collagen.

**Figure 5:**
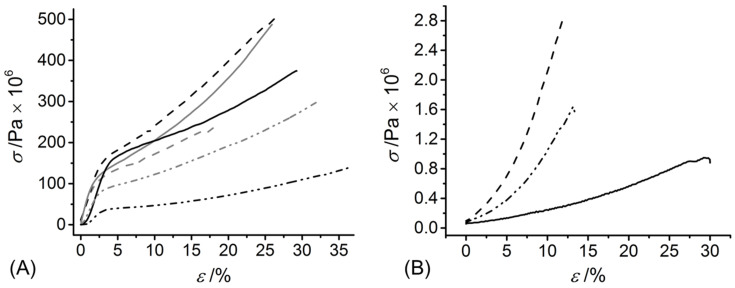
Tensile stress-strain curve of dry (**A**) and hydrated (**B**) wet spun functionalised CRT fibres. (**—**): F-GMA, (**—**): F-GMA*, (**—∙∙—**): F-4VBC, (**—∙∙—**): F-4VBC*, (**— —**): F-4VBC-MA, (**— —**): F-4VBC-MA*.

In line with the 4VBC residues and UV-induced covalent network introduced at the molecular scale, the dry-state *E* and *σ_b_* were higher in both samples of F-4VBC and F-4VBC* (*E* = 3130 ± 600 → 4340 ± 500 MPa; *σ_b_* = 252 ± 40 → 338 ± 50 MPa) compared to the native CRT fibres (*E* = 2189 ± 220 MPa; *σ_b_* = 72 ± 3 MPa), whilst the *ε_b_* did not show any significant change (*ε_b_* = 24 ± 3 − 26 ± 5%) (Table 2 and Figure 5A).

The increased values of *E* and *σ_b_* observed in the UV-cured compared to the wet spun fibres confirm the synthesis of a covalently-crosslinked network at the molecular scale of the fibres. Similar trends were observed with samples of F-GMA*, whilst wet spun fibres F-GMA did not show significant variations compared to the tensile properties of native CRT, indirectly suggesting that the covalent functionalisation with GMA residues enables decreased secondary interactions between methacrylate, compared to 4VBC aromatic residues. Other than samples F-4VBC^(^*^)^ and F-GMA^(^*^)^, sequential functionalisation of CRT with both 4VBC and MA generated wet spun fibres and UV-cured variants with the highest dry-state *E* (4190 ± 500 → 5160 ± 400 MPa) and *σ_b_*, therefore reflecting the nearly complete functionalisation and crosslinking of lysine terminations with both rigid aromatic and flexible aliphatic segments. The values of *E* and *σ_b_* measured on samples F-4VBC* and F-4VBC-MA* were higher than the ones of dry glutaraldehyde-crosslinked wet spun collagen fibres [37] and wet spun composite fibres made of poly(*ε*-caprolactone) and gelatin [41], whilst comparable properties were recorded in dry multifilament collagen yarns [22]. These observations therefore support the validity of the approach presented herein to produce wet spun CRT fibres with tailored mechanical properties, whereby further customisation could be expected by subsequent fibre drawing [39].

Following characterisation in the dry state, attention moved to testing of the CRT fibres following saturation with PBS, as a simple mimetic of biological fluids. In line with previous findings, significantly reduced values of *SR* were measured in PBS-equilibrated fibres F-4VBC-MA* (*SR* = 1880 ± 200 wt.%) with respect to wet fibres F-4VBC* and F-GMA* (*SR* = 3130 ± 300 − 3350 ± 500 wt.%).

These trends in *SR* were reflected by an increase in fibre diameter in all wet samples, whereby sample F-4VBC-MA* was confirmed to display the lowest dimensional variation (Ø = 49 ± 10 → 130 ± 30 µm, Table 2). These swelling-induced increases in fibre diameter were significantly higher than that recorded in glutaraldehyde-crosslinked wet spun collagen fibres with a varying degree of post-spinning drawing [39], whereby a diameter increase percent in the range of 20–80% was measured, in contrast to an increase of at least 165% recorded in sample F-4VBC-MA*.

Despite this very large absorption of water, PBS-equilibrated fibres F-4VBC-MA* displayed an averaged *E* of up to 11 MPa, which was remarkably higher than the values measured for the F-4VBC* and F-GMA* fibres (Table 2 and Figure 5B), in line with the previous trends in compression properties measured on the UV-cured hydrogels (Table 1). The uptake, and consequent plasticising effect [27], of water in the wet spun fibres was also reflected by distinct stress-strain tensile curves in the wet compared to the dry state (Figure 5), whereby an elastic-like behaviour was observed in the PBS-equilibrated samples, in contrast to the multi-step response detected in the dry state. Other than the *E*, the *ε_b_* of swollen samples F-4VBC-MA* (*ε_b_* = 15 ± 4%) was somewhat higher than that sample of F-4VBC* (*ε_b_* = 13 ± 3%), whilst an even higher value was recorded in fibres of F-GMA* (*ε_b_* = 19 ± 5%). This trend may be directly related to the molecular flexibility of the crosslinking segment (methacrylate vs. aromatic) and the crosslink density of the covalent network in respective wet spun fibres.

The above results demonstrated a unique combination of swelling and tensile behaviour in the UV-cured fibres, whereby an averaged *SR* of up to 3350 wt.% was combined with an averaged *E*, *σ_b_* and *ε_b_* of 9 MPa, 1.6 MPa and 13%, respectively. These wet-state tensile properties appeared to be lower than those of drawn wet spun collagen fibres crosslinked with either glutaraldehyde [24,42] or dehydrothermal treatment [25,26,42]. However, other than the effect of fibre drawing, such existing crosslinking methods can be associated with toxicity and clinical safety issues, as well as triple helix denaturation, especially given the high temperature associated with dehydrothermal treatment (110 °C, 5 days).

### 3.5. Fibrillogenesis Study in Wet Spun Fibre

Recapitulation of aligned collagen fibrils via fibrillogenesis ex vivo enables mimicry of specific tissue architectures, e.g., aligned tendons [23], and to introduce an additional experimental dimension to tune the tensile properties of respective collagen material. Self-assembly of collagen triple helices into fibrils has been demonstrated in wet spun CRT samples via fibre incubation in aqueous buffer (37 °C, 24 h) [24,25]. Given that functionalised rather than native CRT was employed in this study, it was of interest to investigate the effect of incubation of respective wet spun fibres in vitro.

**Figure 6:**
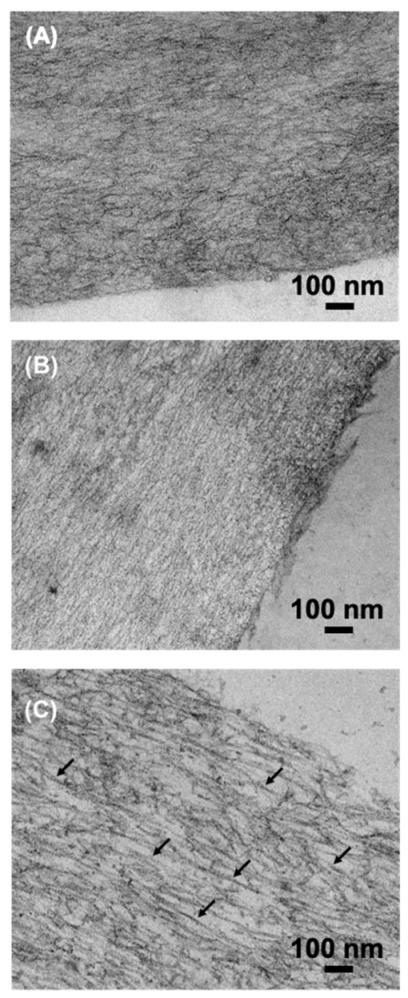
TEM of sample F-4VBC*. (**A**): control F-4VBC* obtained following wet spinning and UV curing (no PBS incubation). (**B**): sample F-4VBC* obtained via sequential wet spinning, 24-h incubation in PBS and UV curing. (**C**): sample F-4VBC* obtained via sequential wet spinning, 72-h incubation in PBS and UV curing. Black arrows indicate re-folded collagen-like fibrils in vitro.

Fibres prepared by sequential wet spinning and UV-curing displayed a random configuration of collagen molecules where no alignment could be detected via TEM (Figure 6A). This observation reflects the absence of a conditioning step in a physiological environment [24,25,26,42] and the increased viscosity of the wet spinning solution of functionalised compared to native CRT [21]. Given its relatively low degree of functionalisation (*F* = 45 mol.%, Table 1), sample F-4VBC was therefore selected and incubated in PBS for up to 72 h (10 mM, pH 7.4, 37 °C) prior to UV curing. An increased degree of alignment was apparent on retrieved samples conditioned for either 24 or 72 h (Figure 6B,C), whereby the incubation time seemed to have a direct effect on fibrillogenesis. While fibres incubated for 72 h displayed some degree of collagen D-banding (black arrows), this effect was not detected on samples incubated for 24 h, whereby an increased degree of alignment was apparent compared to the fibre control.

Given that clear D-periodic collagen fibrils were observed in wet spun CRT fibres following 48-h incubation [24], these TEM images suggest that increased incubation times are required to induce the self-assembly of functionalised collagen molecules, in line with the derivatisation of triple helix-stabilising collagen lysines with photoactive residues, as previously demonstrated by turbidity measurements of respective CRT solutions [27]. Systematic investigations may therefore be required to further elucidate the effect of the specific extent and type of functionalisation on collagen fibrillogenesis.

### 3.6. Cell Tolerability of Fibre Extracts

**Figure 7:**
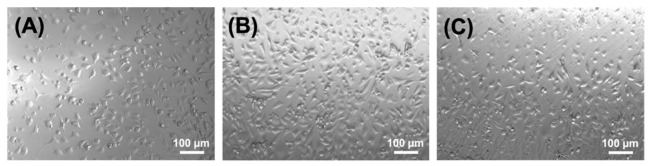
Optical microscopy images of L929 murine fibroblasts following 48-h culture on extracts of wet spun samples F-4VBC* (**A**), F-4VBC-MA* (**B**) and F-GMA* (**C**).

Demonstration of cellular tolerability in vitro is the first step to assess the biocompatibility of a new medical device, e.g., a biomedical membrane. Since UV-cured CRT networks have previously demonstrated to support the viability of L929 fibroblasts [27,28], the attention moved to the testing in vitro of respective wet spun fibres. In contrast to the collagen hydrogel, the microscale diameter of the fibre makes cell seeding and direct cytotoxicity assessment challenging, so 72-h fibre extracts were applied to L929 confluent cells and cell morphology was assessed following 48-h culture. Fibroblasts appeared to display a stretched cell morphology following culture on sample extracts of either F-4VBC*, F-4VBC-MA* or F-GMA*, suggesting active cell proliferation (Figure 7). Interestingly, cell proliferation appeared to be somewhat increased following culture in extracts of F-4VBC-MA* compared to culture in extracts of F-4VBC* and F-GMA*, despite the nearly complete functionalisation of CRT with both 4VBC and MA residues.

These results indicate that fibre extracts are well tolerated by the cells and that risks of delivering unreacted toxic species, e.g., the unreacted photoactive monomers, the photo-initiator or coagulation bath-related residues, are minimal, supporting the high gel content values of the UV-cured collagen networks (Table 1).

## 4. Conclusions

Bespoke photoactive CRT precursors successfully generated hierarchically assembled fibres via sequential wet spinning and UV curing. The type and molar content of the photoactive residues covalently coupled to the CRT backbone enabled varying hydrolytic degradation profiles and compression moduli in respective UV-cured hydrogel networks, as well as retained triple helix configurations. A remarkable combination of macroscopic properties, dependent on the wet spinning photoactive CRT precursor, i.e., high swelling ratio and high wet state tensile modulus, was consequently observed in the wet spun fibres, notably without the need for fibre drawing. Conditioning of the wet spun fibres in physiological conditions enabled a degree of nanoscale alignment and fibrillogenesis, such that collagen-like D-periodic fibrils could be detected.

This new sequential fabrication route of wet spinning and UV curing could therefore be leveraged for the development of three-dimensional collagen membranes customised to the requirements of various healthcare applications, including, e.g., skin repair, guided bone regeneration and minimally invasive surgery.

## Figures and Tables

**Table 1:** Molecular and thermo-mechanical characteristics of UV-cured CRT hydrogels. n.a.: not available.

Sample ID	*F*/mol.-%	*c*/wt.%	*G*/wt.-%	*SR*/wt.-%	*E_c_*/kPa	*σ_b_*/kPa	*ε_b_*/%
4VBC-MA*	93 ± 1	0.8	90	900 ± 180	153 ± 85	112 ± 43	56 ± 4
		0.4	67	1560 ± 100	292 ± 205	190 ± 75	62 ± 7
4VBC*	45	0.8	100	1965	81	n.a.	n.a.
GMA*	65	0.8	100	1262	39	n.a.	n.a.

**Table 2:** Characterisation of functionalised CRT fibres following either wet spinning or UV-curing. Data are presented as the mean ± SD. ^(a)^ PBS-equilibrated samples; n.a.: not available.

Sample ID	Ø/μm	*SR*/wt.-%	*σ_b_*/MPa	*ε_b_*/%	*E*/MPa
F-C	80 ± 3	n.a.	72 ± 3	24 ± 3	2189 ± 220
F-GMA	39 ± 10	n.a.	252 ± 40	26 ± 5	2010 ± 600
F-GMA*	40 ± 10	3130 ± 300	191 ± 50	22 ± 6	3210 ± 900
	150 ± 20 ^(a)^		0.4 ± 0.1 ^(a)^	19 ± 5 ^(a)^	5 ± 1 ^(a)^
F-4VBC	54 ± 5	n.a.	252 ± 40	26 ± 5	3130 ± 600
F-4VBC*	51 ± 10	3350 ± 500	338 ± 50	26 ± 5	4340 ± 500
	140 ± 30 ^(a)^		1.6 ± 0.5 ^(a)^	13 ± 3 ^(a)^	9 ± 1 ^(a)^
F-4VBC-MA	51 ± 5	n.a.	504 ± 30	29 ± 4	4190 ± 500
F-4VBC-MA*	49 ± 10	1880 ± 200	542 ± 80	30 ± 6	5160 ± 400
	130 ± 30 ^(a)^		2.6 ± 0.4 ^(a)^	15 ± 4 ^(a)^	11 ± 4 ^(a)^

## Data Availability

Not applicable.

## Supplementary Materials

The following are available online at https://www.mdpi.com/article/10.3390/membranes11080620/s1, Figure S1: CD spectra of samples of CRT (—), 4VBC (—), GMA (--), 4VBC-MA (···), F-4VBC (—·—), F GMA (--). Figure S2: ATR-FTIR spectrum of wet spun functionalised collagen sample F-4VBC-MA.
