# Supplementary material for: Hierarchically Assembled Type I Collagen Fibres as Biomimetic Building Blocks of Biomedical Membranes
Source: Membranes (Basel). 2021 Aug 12;11(8):620. doi: 10.3390/membranes11080620 (PMC8400969; doi:10.3390/membranes11080620)
Supplement: Supplementary file 1 [file membranes-11-00620-s001.zip › membranes-1323316-supplementary.pdf]

membranes

Supporting Information

Hierarchically Assembled Type I Collagen Fibres as Biomimetic Building Blocks of Biomedical Membranes

Jie Yin [1,2,†], David J. Wood [2], Stephen J. Russell [1] and Giuseppe Tronci [1,2,*]

1 Clothworkers' Centre for Textile Materials Innovation for Healthcare, School of Design, University of Leeds, Leeds LS2 9JT, UK; jie.yin@henkel.com (J.Y.); s.j.russell@leeds.ac.uk (S.J.R.)
2 Biomaterials and Tissue Engineering Research Group, School of Dentistry, St. James's University Hospital, University of Leeds, Leeds LS9 7TF, UK; d.j.wood@leeds.ac.uk
* Correspondence: g.tronci@leeds.ac.uk
† Current address: Henkel (China) Investment Co., Ltd., Shanghai 201203, China.

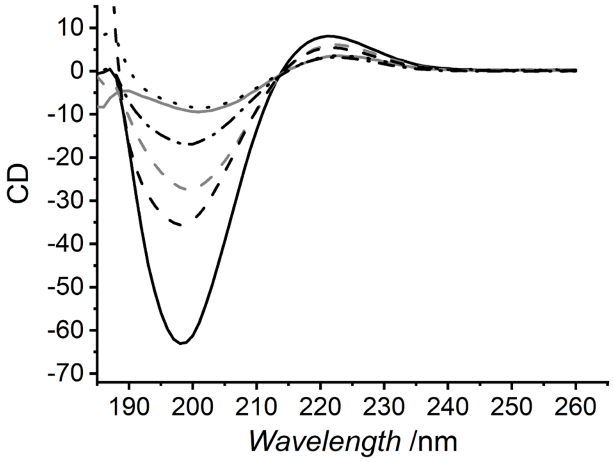

Figure S1. CD spectra of samples of CRT (—), 4VBC (—), GMA (--), 4VBC-MA (···), F-4VBC (—·—), F-GMA (--). .

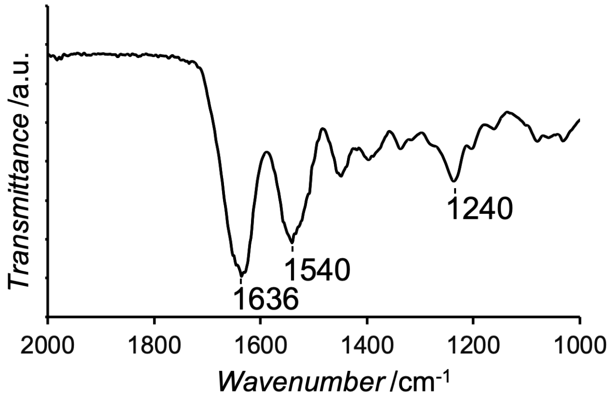

Figure S2. ATR-FTIR spectrum of wet spun functionalised collagen sample F-4VBC-MA.
